# Variation of thermal tolerance during northward range expansion in the invasive golden star tunicate, *Botryllus schlosseri*

**DOI:** 10.1093/conphys/coaf018

**Published:** 2025-04-01

**Authors:** Zachary J C Tobias, Gareth Miller, Carolyn K Tepolt

**Affiliations:** MIT-WHOI Joint Program in Oceanography/Applied Ocean Science and Engineering, Cambridge and Woods Hole, MA, USA; Department of Earth, Atmospheric and Planetary Sciences, Massachusetts Institute of Technology, 77 Massachusetts Avenue, Cambridge, MA 02139, USA; Biology Department, Woods Hole Oceanographic Institution, 266 Woods Hole Road, Woods Hole, MA 02543, USA; Biology Department, Woods Hole Oceanographic Institution, 266 Woods Hole Road, Woods Hole, MA 02543, USA; Department of Environmental Science, Western Washington University, 516 High St, Bellingham, WA 98225, USA; Biology Department, Woods Hole Oceanographic Institution, 266 Woods Hole Road, Woods Hole, MA 02543, USA

**Keywords:** Cardiac physiology, cold performance, heat tolerance, invasive species, LT_50_, tunicate

## Abstract

Populations within a species can differ with respect to their thermal physiology, with variation often observed across gradients in environmental temperature with latitude or elevation. The tempo at which phenotypic plasticity and/or local adaptation are able to shape variation in thermal tolerance has implications for species persistence in an increasingly volatile climate. Having encountered novel environments during introduction and subsequent range expansion, non-indigenous species present useful case studies for examining thermal tolerance differentiation on contemporary time scales. Here we test for differentiation of heat and cold tolerance among three populations of the invasive golden star tunicate, *Botryllus schlosseri* (Pallas), spanning a 24.3° latitudinal gradient in the Northeast Pacific. We observed differentiation of post-larval heat tolerance among our sites, with our southern, putatively warm-adapted population exhibiting a significantly higher LT_50_ than the two more northern populations. We also found that adult cardiac performance at cold temperatures is progressively greater in colder, higher latitude populations. This pattern may suggest compensatory genetic adaptation to colder environmental temperatures. By examining both heat tolerance and cold performance simultaneously among populations of an invasive ascidian, we document how this marine ectotherm is capable of shifting its physiology to novel environmental conditions over compressed time scales, with implications for the spread of this invasive species and, more broadly, for species’ responses to temperature in an era of global change.

## Introduction

Biogeographic ranges often encompass a wide variety of environmental conditions, across which species must tune their physiology to match the local environment. Within species, phenotypic variation is often observed along climatic gradients with latitude or elevation (Hall *et al.*, 2007; Gonzalo-Turpin and Hazard, 2009; Keller *et al.*, 2013; Pereira *et al.*, 2017). Such variation can be shaped by both genetic and environmental factors, through local adaptation and phenotypic plasticity, respectively (West-Eberhard, 2003; Kawecki and Ebert, 2004). Understanding how these processes interact to produce variation in phenotypes is a central goal of many evolutionary studies and can provide insights into the potential for species persistence under an increasingly volatile climate (De Frenne *et al.*, 2013). In contemporary timescales, e.g. in the case of species invasions or range expansions, the establishment of population-level differentiation in key phenotypes such as environmental tolerances hints at the ability for these traits to shift rapidly (e.g. Maron *et al.*, 2004; Monty and Mahy, 2009; Leal and Gunderson, 2012; Carbonell *et al.*, 2021; Neu and Fischer, 2022). This rapid phenotypic divergence across space also speaks to the potential for species to alter their physiology in response to rapid environmental change (Somero, 2010). Until recently, however, intraspecific variation of environmental tolerances, specifically thermal tolerance, has often been overlooked in macrophysiological studies that forecast species responses to climate warming (Valladares *et al.*, 2014; Bennett *et al.*, 2019). An appreciation of intraspecific variation of thermal tolerance and its ability to shift rapidly, then, is critical to understanding species responses to environmental change.

In the oceans, population-level divergence of thermal tolerance is often observed across latitudinal gradients. For marine ectotherms, heat tolerance often increases with decreasing latitude (Zippay and Hofmann, 2010; Sanford and Kelly, 2011; Pereira *et al.*, 2017; Sasaki and Dam, 2019; Sasaki *et al.*, 2022), a presumably adaptive response to warmer environmental temperatures, though invariance of heat tolerance or more complex patterns have also been documented (Kuo and Sanford, 2009; Gaitán-Espitia *et al.*, 2014). While there are comparatively fewer data for latitudinal variation of cold tolerance in marine ectotherms, some studies have revealed similar latitudinal patterns, with more cold-tolerant populations at northern latitudes (Wallace *et al.*, 2014; Chiba *et al.*, 2016; Thyrring *et al.*, 2019). Divergence of cold tolerance can also arise rapidly, as illustrated by Tepolt and Somero (2014), who demonstrated the maintenance of higher heart rates in the cold after a common acclimation temperature in northern populations within the invasive range of the European green crab, *Carcinus maenas*.

Understanding how variation in fitness-related phenotypes is partitioned across space is vital for assessing the capacity for populations and species to adapt in the face of global change (Hoffmann and Sgró, 2011). In recent decades, an increasing number of studies have demonstrated the potential for rapid evolutionary adaptation (e.g. Reznick *et al.*, 1990; Barrett *et al.*, 2011), begging the question of whether adaptation can keep pace with the rate of environmental change (Jump and Peñuelas, 2005; Bradshaw and Holzapfel, 2006). Many such studies have relied on the investigation of non-indigenous species (NIS) (Gilchrist *et al.*, 2004; Maron *et al.*, 2004; Colautti and Barrett, 2013; reviewed by Colautti and Lau, 2016). As NIS, by their very nature encounter new habitats during introduction, they present useful ‘natural experiments’ for studying species responses to novel environmental conditions on contemporary timescales (Huey *et al.*, 2005; Sax *et al.*, 2007). For example, in the common Puerto Rican anole, *Anolis cristatellus*, cold tolerance in the more cold-exposed invasive population in Florida is significantly greater than in the native range, with this differentiation arising on the order of just ~35 generations (Leal and Gunderson, 2012). The pace at which NIS are able to adapt to new habitats thus provides insights into the ‘best case scenario’ for how native species may respond to various axes of anthropogenic change (Moran and Alexander, 2014).

One consequence of global climate change is the observed poleward range shift/extension of many species (Sorte *et al.*, 2010a; Chen *et al.*, 2011; Pinsky *et al.*, 2020). While this is perhaps most obviously driven by an increase in temperatures at higher latitudes, there are also more complex drivers. For example, in the marine realm there is an increasing concern about introductions of NIS to northern latitudes because of a predicted increase in global shipping in the Arctic due to retreating sea ice (Ware *et al.*, 2016; Chan *et al.*, 2019). Due to shipping and other modes of dispersal, northward spread of NIS may outpace the rate of warming, provided that these species have the physiological capacity to deal with colder northern temperatures. Many NIS exhibit intrinsically broad physiological tolerances (Zerebecki and Sorte, 2011; Higgins and Richardson, 2014) and may thus be primed for success at northern latitudes (Kelley *et al.*, 2013; Tepolt and Somero, 2014).

One high-profile marine NIS that is currently expanding its range northward is the golden star tunicate, *Botryllus schlosseri* (Pallas). *Botryllus schlosseri* is a colonial ascidian and a common member of fouling communities, typically growing on the bottoms of docks, boat hulls and other substrates within harbours and marinas. While its native range is unknown, *B. schlosseri* is invasive across much of the global ocean, including parts of the North Atlantic, South Pacific and Northeast Pacific (Fofonoff *et al.*, 2024). In North America, it is actively expanding northward along both coasts (Callahan *et al.*, 2010). On the west coast of the United States, *B. schlosseri* was first detected in San Francisco Bay in 1947 (Carlton, 1979) and has since spread southward as far as San Diego, CA, where it was detected in 1965 (Lambert and Lambert, 1998), and northward as far as Sitka, AK, where it was detected in 2001 (Ruiz *et al.*, 2006). Globally, its most recent northern range expansion was reported in Iceland, where it was found in 2011 (Ramos-Esplá *et al.*, 2020). This global northward range expansion into cooler waters, along with its persistence at lower latitudes (Ali *et al.*, 2009; Tovar-Hernández *et al.*, 2014), demonstrates that *B. schlosseri* is capable of inhabiting a broad range of thermal habitats.

**Figure 1:**
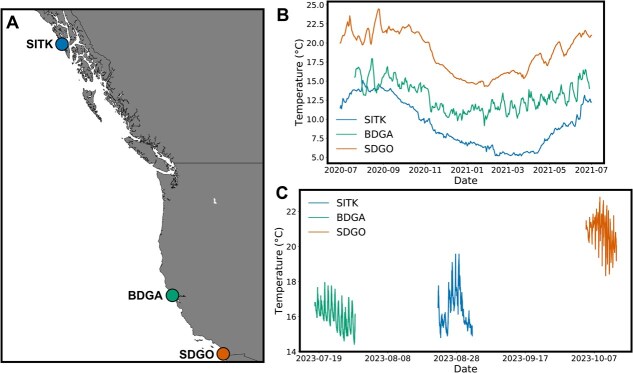
(**A**) Map of study sites. SITK = Sitka, AK, USA. BDGA = Bodega Bay, CA, USA. SDGO = San Diego, CA, USA. (**B**) Mean daily environmental temperatures at the three sites from July 2020 to July 2021. (**C**) *In situ* temperatures recorded during experimentation periods in the summer/fall of 2023.

There has been limited exploration of the potential for population-level divergence of thermal tolerance in *B. schlosseri*. We previously found population-level differentiation of post-larval heat tolerance across a thermal gradient in the Northwest Atlantic (Tobias *et al.*, 2024). Here, we compare *B. schlosseri* thermal tolerance in its invasive range in the Northeast Pacific, focusing on three populations spanning a thermal gradient encompassing 24.3° of latitude. We assess population-level differentiation of both heat tolerance, through LT_50_ experiments on newly settled post-larvae, and cold performance, through a cardiac assay during a cold challenge in adult *B. schlosseri*. We find that populations differ in their sensitivity to heat, with northern populations exhibiting lower thermal tolerance than their southern counterparts. For cold performance, we observed that northern populations maintained progressively higher heart rates in the cold after acclimation at a common temperature. By comparing thermal tolerance across populations, we illustrate that this species may be able to tune its physiology to local habitats on a contemporary timescale, providing important insights into the capacity of marine organisms to adapt to changing environmental conditions.

## Materials and Methods

### Sites

We conducted LT_50_ and cardiac performance experiments on *B. schlosseri* individuals collected from three sites spanning 24.3° of latitude in the Northeast Pacific: Eliason Harbor, Sitka, AK, USA (SITK); Bodega Harbor, Bodega Bay, CA, USA (BDGA); and Sea World Marina, San Diego, CA, USA (SDGO) (Fig. 1A, Table 1). Experiments at SITK were performed at the Sitka Sound Science Center, BDGA at the University of California at Davis’ Bodega Marine Lab, and SDGO at San Diego State University’s Coastal and Marine Institute Laboratory.

**Table 1:** Site information and heat tolerance experiment data, with lower and upper 95% confidence limits around LT_50_

Location	Code	Lat	Lon	Oozooids	Clutches	LT_50_ (°C)	Lower	Upper
San Diego, CA	SDGO	57.058	−135.354	535	14	32.28	32.20	32.37
Bodega Bay, CA	BDGA	38.329	−123.057	338	24	31.52	31.39	31.64
Sitka, AK	STIK	32.767	−117.231	148	10	31.54	31.40	31.68

### Environmental temperature

*In situ* temperatures were recorded at the collection sites during the 2-week experimentation periods using HOBO Pendant temp/light loggers (Onset, Bourne, MA, USA; cat. no. UA-002-64). Long-term temperature for stations near our collection sites was obtained from publicly available sources for SDGO (NOAA, 2023a) and SITK (NOAA, 2023b). For BDGA, temperature data for 2020–21 were generously shared by Dr Jay Stachowicz.

### LT_50_ experiments

We conducted LT_50_ experiments as described previously (Tobias *et al.*, 2024), with some modifications. Two days prior to each experiment ~50 *B. schlosseri* colonies were collected haphazardly by hand from the underside of floating docks. Collections occurred at each site during the following time spans: BDGA, 18–26 July 2023; SITK, 22–29 August 2023; SDGO, 3–9 October 2023. Colonies were transported to the laboratory and placed in groups of 2–3 in 3.5 × 2 × 2-inch wells of polycarbonate compartment boxes with ~200 ml of local seawater. Five extra-thick microscope slides (Fisherbrand, Pittsburgh, PA, USA; cat. no. 22–267-013) were placed along the bottom and side walls of each compartment, to allow for larval settlement upon the slides. Colonies were then kept at room temperature over 2 nights, exchanging seawater twice during the first day after collection and once the second day after collection. Once slides were observed to be covered in settled larvae, we transferred them to a separate holding tank with aeration. Two days after collection, all oozooids (metamorphosed settled larvae) were censused under a Stemi dissecting stereomicroscope (Zeiss, Oberkochen, Germany) and examined for a heartbeat and normal development. Those with slowed (tail still present) or abnormal development were removed, as well as those settled on the extreme margin of the slides. Slides were further thinned of oozooids to retain a maximum of 35 individuals.

Up to five slides from each clutch (offspring of a single colony, as we never observed multiple colonies from one compartment simultaneously release larvae) were partitioned across five temperature treatments. In SITK and BDGA, the set temperatures were 29.4, 30.0, 30.6, 31.1 and 31.7°C, whereas at SDGO the set temperatures were 30.0, 30.6, 31.1, 31.7 and 32.2°C. We selected these temperatures based on the results from Tobias *et al.* (2024), with the expectation that SDGO would exhibit a higher LT_50_, thus the use of a slightly higher temperature range. The heat tolerance assays were performed in 2 l of seawater in polycarbonate 4-inch-deep one-third pans (Cambro, Huntington Beach, CA, USA; cat. no. 34CW) equipped with programmable 25 W glass aquarium heaters (YOFOTHS, Shenzhen, Guangdong, China) and HOBO pendant light/temp temperature loggers. Temperatures used for downstream analysis were those recorded by the HOBO loggers rather than the set temperatures on the heaters.

Heat exposures lasted 20 h, starting from the ambient temperature of local seawater. The ramp speed was ~4°C/h, chosen to conform with previously conducted experiments of *B. schlosseri* populations in the Northwest Atlantic (Tobias *et al.*, 2024). After 20 h, the oozooid slides were removed and live oozooids (those with a detectable heartbeat) were censused.

### Cardiac performance experiment

To assess the potential for variation in cold performance among the three populations, we conducted an assay of adult *B. schlosseri* cardiac performance during a cold challenge. We used the same colonies as collected for the LT_50_ experiment. If kept undisturbed, *B. schlosseri* colonies will often begin to attach to and grow out along the bottom slide within each well of the compartment box. We treated the colonies as described above, performing two water exchanges the day after collection and one water exchange 2 days after collection. If 2 days after collection a colony was firmly attached to the slide, we transferred it to a holding tank of room temperature seawater with aeration overnight, placing it vertically in a slide storage box. On the third day after collection, we placed colonies in a portable incubator (IVYX Scientific, Seattle, WA, USA) set to 18°C for 24 h. By acclimating individuals from different populations to a common temperature, we sought to minimize the potential effect of short-term phenotypic plasticity on the outcome of the cardiac performance experiment. We chose 18°C as a common acclimation temperature, as this was within the range of temperatures experienced in the field at all sites (Fig. 1C).

After the 24-h acclimation (on the fourth day after collection), colonies were subjected to a cold challenge experiment. Slides with colonies were placed in a specialized imaging chamber designed and 3D-printed at Woods Hole Oceanographic Institution on an Objet350 Connex3 3D printer (StrataSys, Eden Prairie, MN, USA). Seawater was pumped to this chamber from an insulated sump tank (Coleman Party Stacker cooler, Chicago, IL, USA; cat. no. 3000005591) using a small aquarium pump (Hygger, Shenzhen, China; cat. no. HG-939). An additional pump (Hawthorne Hydroponics EcoPlus, Vancouver, WA, USA; cat. no. HGC728310) fed water to a one-thirteenth horsepower seawater chiller (AquaEuro USA, Los Angeles, CA, USA; cat. no. AC13H). We converted the chiller to on/off operation by soldering together two circuits in the control panel to bypass the chiller’s temperature controller. Temperature was controlled using an external programmable temperature controller (Bayite, Zhongshan, China; cat. no. BTC201). In case ambient seawater was <18°C, we also included a 300 W aquarium heater (Finnex, Countryside, IL, USA; cat. no. TH-300S) in the sump. We included a HOBO pendant temp/light logger in the sump to record experimental temperatures. An image of the experimental set-up is included in Supplementary Fig. 1.

Starting from 18°C, we ramped down the temperature to 2°C over the course of an hour, for a ramp speed of ~16°C/h. During this ramp, we recorded a video of one to several zooids’ (individuals within the colony) hearts under an M80 stereomicroscope (Leica, Wetzlar, Germany) fitted with a microscope camera (AmScope, Irvine, CA, USA; cat. no. MU1803-HS). Videos were acquired using the AmLite software (AmScope), adding a timestamp. To prevent condensation on the imaging chamber at cold temperatures, we added a thin layer of fresh water to the upper surface of the outside of the chamber. At the end of each experiment, *B. schlosseri* colonies were discarded.

Heart rates over time were extracted from the videos manually. Starting from the first full minute according to the video timestamp, we counted the number of heartbeats per minute at 5-min intervals. If the heartbeat was arrhythmic or there were issues with the video quality (out of focus, out of frame, etc.), we skipped that minute and proceeded to subsequent minutes until conditions improved. For videos that contained footage of multiple hearts, we counted each heart separately but kept track of colony membership to account for potential colony effects during analysis. As *B. schlosseri* colonies cyclically undergo replacement of adult zooids by buds, we excluded all zooids in blastogenetic stage D (i.e. those not actively syphoning); these are known to exhibit aberrant physiology (pers. comm., A. Voskoboynik).

### Data analysis

For the LT_50_ experiment, analyses were performed in R v. 4.1.2 (R Core Team, 2013). We used the R package *drc* v. 3.0.1 (Ritz *et al.*, 2015) to estimate LT_50_ values for each population, fitting two-parameter (slope and midpoint) log-logistic models to each population’s binomial survivorship data. To test if there was a significant effect of population on LT_50_, we compared a model with separately estimated LT_50_ values for each population to a null model with a jointly estimated LT_50_ value for all populations using a likelihood ratio test. *Post hoc* pairwise comparisons among populations were conducted using the same approach, with *P*-values adjusted using the Bonferroni method to account for multiple comparisons (Tobias *et al.*, 2024).

For the cardiac performance experiment, analyses were performed in Python v. 3.11.5 (Van Rossum and Drake, 2009). Heart rate over time was converted to heart rate over temperatures using the temperature recorded by the sump tank HOBO logger at the start of each minute. We compared the heart rate at 18°C, the heart rate at 8°C, and the Q_10_ in heart rate between these two values among all populations. Because many heart rate records did not include values for heart rate at the exact temperatures of 18 and 8°C, we used scikit-learn v. 1.2.2 (Pedregosa *et al.*, 2011) to perform linear interpolation, extracting predictions for these values. To ensure we were not extrapolating outside the bounds of the available data, we excluded all records that did not span the full 8–18°C range.

To test for differences among populations in these values (heart rate at 18°C, heart rate at 8°C and Q_10_), we performed linear mixed effects modelling using the Python package statsmodels v. 0.14.0 (Seabold and Perktold, 2010). To test for an effect of population on these responses, we included population as a fixed effect and colony membership as a random effect. We chose to include colony membership as a random effect because there is the potential for differences in heart rate to be driven by colony-level phenomena (genetic differences, blastogenetic stage, etc.), and we were primarily interested in differences among populations. Because we hypothesized that there would be an ordering in these values according to population latitude (SDGO expected to exhibit the lowest heart rate at 8°C, followed by BDGA, then SITK), we encoded population according to an ordinal scale, with SDGO assigned a value of 0, BDGA a value of 1 and SITK a value of 2. To assess how much variation in the response variables can be attributed to colony membership, we calculated the intra-class correlation coefficient (ICC) by dividing the variance explained by colony membership by the sum of itself and the unexplained variance in the model (‘scale’ value in model output). To perform *post hoc* pairwise comparisons among populations, we repeated the linear mixed effect modelling on groupings containing data for population pairs, adjusting *P*-values using the Bonferroni method to account for multiple comparisons.

### Ethical declarations

All *B. schlosseri* collections and experiments were conducted under the following permits: California Department of Fish and Wildlife S-230510002-23 062-001 and Alaska Department of Fish and Game CF-23-037.

## Results

### Environmental temperature

As expected, environmental temperature varied strongly with latitude (Fig. 1B, Supplementary Tables 1–3). However, at certain times during the summer of 2020, mean daily temperatures overlapped between BDGA and SITK. Similarly, during the experimentation periods in the summer of 2023 we observed overlap between the *in situ* temperatures at BDGA and SITK (Fig. 1C).

### Heat tolerance

**Figure 2:**
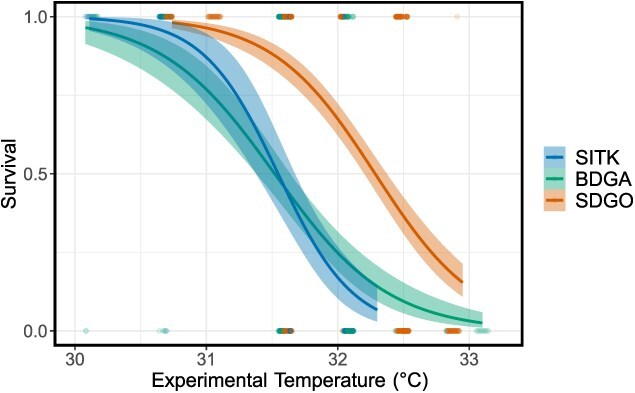
Differentiation of heat tolerance, with SDGO exhibiting significantly greater heat tolerance than SITK and BDGA. Points represent individual survivorship data with lines illustrating logistic regressions of these data. Shaded areas around each line represent 95% confidence interval.

In total between the three sites, 1021 oozooids from 48 clutches were included in the experiment (Table 1). Logistic survivorship curves illustrate how heat tolerance varied according to population (Fig. 2). LT_50_ estimates for SITK, BDGA and SDGO were 31.54, 31.52 and 32.28°C, respectively (confidence intervals provided in Table 1). A model with separately estimated LT_50_ values for each population explained the data significantly better than a model including a jointly estimated LT_50_ value for all populations (𝜒^2^(2) = 132.86, *P* < 0.001). *Post hoc* comparisons between pairs of sites revealed significant differences between SDGO and SITK (*P* < 0.001) and SDGO and BDGA (*P* < 0.001), but not between SITK and BDGA (*P* = 1), as illustrated in Fig. 2.

### Cold performance

**Figure 3:**
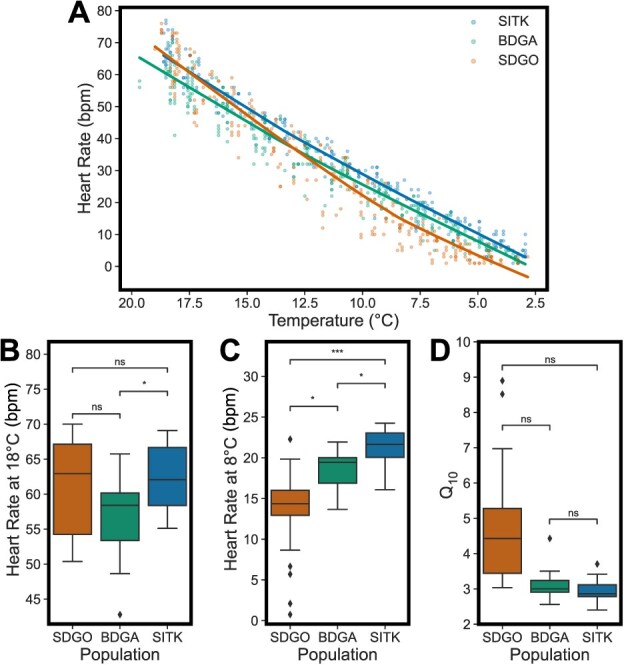
Populations differ in cardiac performance during a cold challenge. (**A**) Heart rates during the course of the experiments. Points represent heart rate in a zooid at a given experimental temperature. Lines are locally weighted scatterplot smoothing (LOWESS) fits to each population’s heart rate data. The *x*-axis is reversed to reflect the progression of the experiments. (**B**) Boxplot of interpolated heart rates at 18°C by population. (**C**) Boxplot of interpolated heart rates at 8°C by population. (**D**) Boxplot of Q_10_ values between at 8 and 18°C by population. Two outlier values not displayed for SDGO (24.37 and 69.69) due to *y*-axis limits. Significance testing between pairs of populations was carried out by linear mixed effect modelling, with *P*-values adjusted via the Bonferroni method. ^*^P_adj_ < 0.05, ^**^P_adj_ < 0.01, ^***^P_adj_ < 0.001, ns = non-significant.

**Figure 4:**
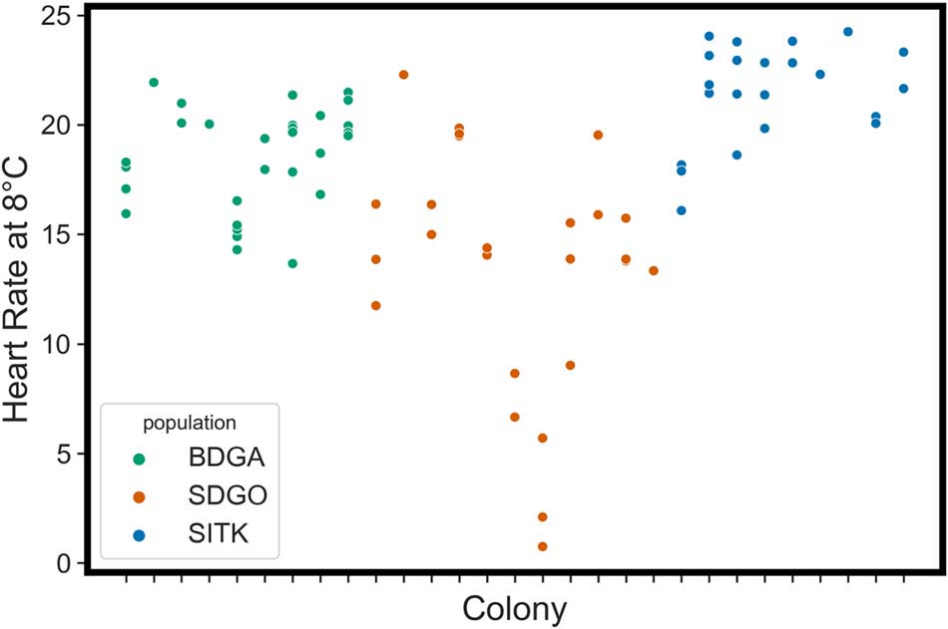
Interpolated heart rate at 8°C of zooids by colony membership

Among the three sites, we obtained heart rate records for 86 zooids from 31 colonies. Overall, heart rate decreased with decreasing temperatures over the course of the experiment (Fig. 3A). After removing records that did not span the full 8–18°C temperature range, 81 zooids from 29 colonies remained (9–11 per site). Linear mixed effect modelling revealed a significant effect of population on heart rate at 8°C (*P* < 0.001), but not heart rate at 18°C (*P* = 0.684) or Q_10_ (*P* = 0.0875). Colony membership was observed to have a strong effect on interpolated heart rate at 8°C, with an ICC of 0.809 (Fig. 4). *Post hoc* pairwise comparisons revealed significant differences in interpolated heart rate at 18°C between SITK and BDGA (*P*_adj_ = 0.0262), but not between SITK and SDGO (*P* = 1) or BDGA and SDGO (*P*_adj_ = 0.294) (Fig. 3B). Interpolated heart rate at 8°C followed a latitudinal pattern, with significantly higher heart rates in SITK than BDGA (*P*_adj_ = 0.0114), significantly higher heart rates in BDGA than SDGO (*P*_adj_ = 0.0196) and correspondingly significantly higher heart rates in SITK than SDGO (*P*_adj_ < 0.001) (Fig. 3C). Q_10_ similarly followed a latitudinal pattern but reversed, with higher values at lower latitudes (Fig. 3D). However, all pairwise comparisons for Q_10_ were non-significant, likely due to high variability in SDGO.

## Discussion

Whether and how species will be able to adapt to rapidly changing environmental conditions is a central question in the contemporary study of ecology, evolution and conservation (Hoffmann and Sgró, 2011). Because they experience novel conditions during introduction, NIS present excellent case studies for understanding modalities of adaptive responses, through local adaptation or phenotypic plasticity(Matesanz *et al.*, 2010; Moran and Alexander, 2014). In the present study, we assessed phenotypic differentiation of heat tolerance and cold performance across a latitudinal gradient during range expansion in the invasive golden star tunicate, *B. schlosseri*. We demonstrate that colder, more northern populations exhibit lower heat tolerance than a warmer, southern counterpart. For cold performance, we uncovered a pattern of northern populations progressively maintaining higher heart rates in cold temperatures after a common acclimation treatment. Together, our results illustrate how a widespread invasive species may have shifted its tolerance to better match the local thermal environment, providing insights into the ability of species to adapt to changing environmental conditions on contemporary timescales.

### Population differentiation of heat tolerance

We found that the more northern populations of SITK and BDGA exhibited lower LT_50_ values than the southern population of SDGO (Figs 2 and 3), indicating that they possess lower heat tolerances. Given the prevailing environmental temperatures (Fig. 1A), we expected to observe a gradient in LT_50_ (SITK < BDGA < SDGO). However, we observed no significant difference between SITK and BDGA. This observation could be due to a number of factors. First, while mean annual temperatures are lower in SITK than BDGA, temperatures at the two sites during the summer are quite similar. For example, in August 2020 local temperatures in BDGA were actually lower than in SITK (Fig. 1B) despite the large difference in latitude. We observed this from our own temperature records for 2023 as well, where the mean temperature for 2 weeks in late July in BDGA was lower than for 2 weeks in late August/early September in SITK (Fig. 1C). Coastal upwelling, which is prominent along the central California coast, may explain this lack of a strong latitudinal gradient in summer temperature (Huyer, 1983). Thus, because *B. schlosseri* are most reproductively active in the summer (Stewart-Savage *et al.*, 2001; Epelbaum *et al.*, 2009), the selective gradient for heat tolerance in early development between these two sites might simply not exist. Alternatively, or perhaps additionally, the similarity in heat tolerance between BDGA and SITK could be driven by phenotypic plasticity. We previously demonstrated that the temperature during development has a strong effect on heat tolerance after settlement, with higher developmental temperatures driving greater heat tolerance (Tobias *et al.*, 2024). Because the thermal environment the oozooids were experiencing as embryos did not differ substantially between SITK and BDGA (Fig. 1C), the similarity in LT_50_ may have been an effect of similar environments. Despite the lack of a difference between SITK and BDGA, the observation of a higher LT_50_ in SDGO still provides evidence for differentiation of heat tolerance in the direction we anticipated.

Whether this differentiation of heat tolerance is driven by divergence within the invasive range since *B. schlosseri*’s initial introduction is unclear, however. Genomic data for this species indicates that there have been at least two introductions to the west coast of North America, with populations from southern California forming a lineage that is evolutionarily distinct from populations further north (Tobias, 2024). These separate lineages may differ physiologically due to variation that evolved with different source populations rather than shifting after introduction. Thus, the differentiation of heat tolerance that we observe here could be a signature of pre-adaptation, where a putatively warm-adapted source seeded the introduction to southern California and a more heat-susceptible population was the source of introductions further north. Because the ongoing genomic study sampled strictly from North America, it is not able to address the potential origins of *B. schlosseri* on the west coast, precluding a more detailed examination of pre-adaptation. Nonetheless, this potential scenario draws parallels to the invasion of the east coast of North America by the European green crab, *C. maenas*. Its original introduction to New England in the early 1800s is likely to have been a from a south–central European source (Roman, 2006). While northward spread was originally confined to the Gulf of Maine (Carlton and Cohen, 2003), further expansion in recent decades coincided with the arrival of a second introduction from a northern European, cold-adapted source (Roman, 2006; Tepolt and Somero, 2014).

With the caveat that adaptive differentiation may have arisen prior to introduction, the observation of higher heat tolerance in the southern population of SDGO is consistent with local adaptation to temperature. However, because the individuals used in the LT_50_ experiments were not reared in a common garden, it is not possible to disentangle genetic and environmental effects. As noted above, *B. schlosseri* oozooids possess appreciable levels of developmental plasticity for heat tolerance. Tobias *et al.* (2024) showed that for every 1°C increase in developmental temperature, LT_50_ can increase by as much as 0.187°C, depending on the population. The difference in mean temperature during the experimentation periods between our coldest site (BDGA, 16.03°C) and our warmest site (SDGO, 20.87°C) was 4.84°C. Thus, if we assume an acclimation response ratio (ARR) of 0.187°C/1°C developmental temperature, we would expect a maximum difference in LT_50_ of 0.91°C between BDGA and SDGO. That we observe a difference of 0.76°C indicates that the differentiation in LT_50_ could be due solely to phenotypic plasticity. This is of course predicated on the assumption that populations on the west coast of North America harbour similar levels of developmental plasticity as the most plastic population investigated on the east coast (Sandwich, MA, USA; SAND). We previously demonstrated that in addition to exhibiting differentiation of heat tolerance, east coast populations differ in their degree of developmental plasticity and that this appears to be related to the extent of short-term temperature variability, with more variable sites exhibiting greater levels of developmental plasticity. Given that the west coast populations experience lower levels of daily variability (Fig. 1C here versus Fig. 1c and Fig. S5 of Tobias *et al.* (2024)), we might expect them to possess lower ARRs and thus a smaller predicted difference in LT_50_ than was observed, allowing for some role of genetics in shaping this differentiation. Consistent with this, our ongoing genomic study of North American *B. schlosseri* populations revealed strong genetic differentiation and high genetic diversity, increasing the likelihood of local adaptation (Tobias, 2024). Ultimately, it is likely that both genetics and the environment shape this observed variation. Future studies should employ a multigenerational common garden approach to fully distinguish between the contributions of local adaptation and phenotypic plasticity in shaping the population differentiation (or lack thereof) that we observe between the three populations (Tobias *et al.*, 2024).

Heat tolerance is a critical trait for species persistence in a rapidly changing ocean (Somero, 2010; Pinsky *et al.*, 2019). Mean sea surface temperature is increasingly rapidly across much of the global ocean (Cheng *et al.*, 2022), and this is compounded by the increasing frequency of more local marine heatwave events (Oliver *et al.*, 2021). Understanding how species may be able to shift their heat tolerance is thus of vital importance for making predictions of range shifts, local extirpations and even species extinction (Somero, 2010; Sunday *et al.*, 2012; Diamond, 2017; Morley *et al.*, 2019). By examining geographic variation of thermal tolerance as a lens through which to view temporal responses to climate warming, a space-for-time approach (Pickett, 1989), while controversial (Damgaard, 2019; Perret *et al.*, 2024), may indicate which populations may be at risk and whether a species has the capacity to shift its physiology to match future environmental conditions (Sorte *et al.*, 2011; Blois *et al.*, 2013; Lovell *et al.*, 2023). The patterns of heat tolerance observed in the present study and previously (Tobias *et al.*, 2024) illustrate how this critical phenotype varies across space, and how it may be shaped by both genetics and environment. Given *B. schlosseri*’s flexibility and its potential to shift its heat tolerance towards the prevailing environmental temperature (Fig. 3), we may conclude that it is poised to be among the ‘winners’ in an era of climate change (Somero, 2010). Other studies have indicated that invasive ascidians may fare comparatively well under a regime of increasing ocean temperatures (Sorte *et al.*, 2010b; Dijkstra *et al.*, 2011; Zhang *et al.*, 2020), demonstrating synergistic interactions between two key axes of global environmental change—species introductions and climate change (Hellmann *et al.*, 2008; Rahel and Olden, 2008; Mainka and Howard, 2010).

### Variation of cardiac function at cold temperature

We found that sites also differed in their cardiac performance at cold temperatures, with more northern populations exhibiting progressively higher heart rates at 8°C (Fig. 3C). The heart rate of ectotherms is inextricably linked to environmental temperatures, with cooler temperatures driving lower heart rates. In the absence of any genetic effects, one might expect to observe lower heart rates in the colder, more northern populations, a pattern driven solely by the prevailing environmental temperatures. However, that we observe the reverse pattern after a common acclimation treatment suggests that compensatory genetic adaptation to cold temperatures may contribute to this pattern.

In contrast to our heat tolerance results, we demonstrate that the pattern for cold performance is clinal along the latitudinal gradient, with SITK possessing the greatest performance in the cold, followed by BDGA and then by SDGO. It is worth noting that this may be an effect of experimenting on adult animals for cold performance in contrast to post-larvae (oozooids) for heat tolerance. As noted above, summer temperatures were overlapping between SITK and BDGA, potentially driving the lack of differentiation in heat tolerance we observed between these two sites. Because oozooids are only present in the summer and early fall, when temperatures at the sites are similar, there may be no selective pressure for divergence of heat tolerance. For cold performance, on the other hand, we experimented on adults, which overwinter. Mean annual temperatures, and especially winter temperatures, do indeed follow a gradient with latitude (Fig. 1B). Thus, in the case of cold performance, there may be differential selection among the populations, driving the pattern of variation we observed.

While animals were acclimated to a common temperature of 18°C, it is possible that lingering environmental effects contributed to the observed pattern. We previously demonstrated developmental plasticity of heat tolerance (Tobias *et al.*, 2024), and it is conceivable that increased cold performance could be conveyed by colder developmental temperatures as well. Because we could not control the juvenile environment, we were not able to fully separate what might be a plastic response from a presumed genetic adaptation. Further, as phenotypes can be shaped by parental or even grandparental environments through transgenerational plasticity (Shama and Wegner, 2014; Donelson *et al.*, 2018), without multigenerational experiments it is impossible to definitively prove that local adaptation contributes to the observed pattern. However, there are a great number of studies that use field-collected, lab-acclimated organisms to infer the potential for evolutionary divergence of thermal physiology (e.g. Tepolt and Somero, 2014; Chiba *et al.*, 2016; Thyrring *et al.*, 2019; Broitman *et al.*, 2021). These approaches are especially important for the most non-model of species that lack tractable methods for intergenerational culture.

While we cannot rule out that phenotypic plasticity may be a contributing factor, other lines of evidence bolster the case for genetic adaptation. We observed that in addition to variation in cold performance among populations, there is substantial variation among colonies ‘within’ populations. This likely has a genetic basis, as individuals from the same colony tended to express more similar cardiac responses to cold than individuals from other colonies (Fig. 4), as indicated by the high ICC. This observation strongly suggests the existence of substantial genetic variation upon which natural selection could act to drive evolutionary divergence of cold performance among populations. Furthermore, as with differentiation of heat tolerance, genomic data can provide additional insights into *B. schlosseri*’s potential for thermal adaptation. Populations from San Francisco Bay and northward to Sitka appear to form a single evolutionary lineage that is distinct from populations in southern California (Tobias, 2024). That we observe differentiation of cold performance between SITK and BDGA, which are evidently within the same clade, indicates that this divergence likely arose during range expansion. Given that *B. schlosseri* was first detected in northern California in 1947 (Carlton, 1979) and was only found in Sitka in 2001 (Ruiz *et al.*, 2006), this speaks to the rapid pace at which evolutionary adaptation may occur. Lastly, several characteristics of *B. schlosseri* point towards the high potential for local adaptation, including strong population genetic differentiation (Grosberg, 1987; Yund and O’Neil, 2000), pronounced genetic diversity (Tobias, 2024) and high mutation rates in ascidians (Tsagkogeorga *et al.*, 2012; Berna and Alvarez-Valin, 2014). Similar to heat tolerance, we recommend that future studies utilize a multigenerational approach to fully disentangle the contributions of genetic and environmental effects on the differentiation of cold performance.

In contrast to studies of heat tolerance, there have been comparatively fewer studies that examine intraspecific geographic patterns of cold performance/tolerance in marine ectotherms (but see Jansen *et al.*, 2007; Tepolt and Somero, 2014; Wallace *et al.*, 2014; Thyrring *et al.*, 2015, 2019; Broitman *et al.*, 2021; Dwane *et al.*, 2023 and others), perhaps due in part to the difficulty of conducting such experiments but also given the relevance of assessing heat tolerance in an era of climate warming. Similar to our findings, Dwane *et al.* (2023) found that individuals of the intertidal snail *Littorina saxatilis* from northern populations maintained higher heart rates at cold temperatures than their more southern counterparts. Higher physiological rates among populations at higher latitudes or elevations have been observed in a diverse array of organisms (Conover and Present, 1990; Schultz and Conover, 1997; Milla *et al.*, 2009; Kaluthota *et al.*, 2015; Storz *et al.*, 2019) and is a central component of the metabolic cold adaptation (MCA) hypothesis (Krogh, 1916; Scholander *et al.*, 1953; Wohlschlag, 1964). While the MCA hypothesis remains controversial (Pörtner *et al.*, 2006), as investigation has revealed some confirmatory (Addo-Bediako *et al.*, 2002; White *et al.*, 2012) but largely contradictory evidence (Clarke and Johnston, 1999; Harper *et al.*, 2000; Lardies *et al.*, 2004; Messamah *et al.*, 2017), it remains a useful theoretical framework for testing how key physiological parameters vary across thermal gradients. While we did not measure metabolic rate in the present study, heart rate is often closely linked to metabolism in both ectotherms and endotherms (Green *et al.*, 2001; Green, 2011; Bruning *et al.*, 2013; Kinoshita *et al.*, 2022), allowing us to interpret our results in the context of MCA. In contrast to many studies investigating MCA in marine ectotherms (Holeton, 1974; Clarke and Johnston, 1999; Sokolova and Pörtner, 2003; but see Tepolt and Somero, 2014; Thyrring *et al.*, 2015; Dwane *et al.*, 2023), we found evidence of latitudinal compensation, whereby northern, putatively cold-adapted populations maintained higher heart rates at a common cold reference temperature than populations further south. Again, whether this truly represents evolutionary adaptation or long-term phenotypic plasticity is unresolved. Nonetheless, that we observe this pattern in *B. schlosseri* is in accordance with MCA, reinforcing the notion that while MCA may not be the rule, it should not be discounted entirely (Pörtner *et al.*, 2007).

### Thermal limits, species invasions and responses to novel environments

Temperature is a fundamental abiotic factor shaping ecological niches (Hochachka and Somero, 2002), and understanding species’ thermal breadths, and to what extent they are flexible, is critical to predicting responses to global climate change (Chown *et al.*, 2010; Sunday *et al.*, 2012). By examining both heat tolerance and cold performance in the same set of populations, we demonstrate how *B. schlosseri* has been able to shift its thermal physiology in response to prevailing local environmental temperature. Further, by assessing differentiation of thermal tolerance in an NIS, we can make inferences about how local adaptation and/or phenotypic plasticity are able to drive divergence of thermal tolerance on contemporary time scales.

We observed that northern, putatively cold-adapted populations are more sensitive to heat stress and tolerant of cold, whereas southern, putatively warm-adapted populations are more tolerant of heat and sensitive to cold. The negative correlation between heat tolerance and cold performance indicates that there may be costs associated with maintaining thermal tolerance in the non-selective environment. Trade-offs between heat and cold tolerance have been observed in other studies (Bennett and Lenski, 2007; Rodríguez-Verdugo *et al.*, 2014; Schou *et al.*, 2022; Xiao *et al.*, 2024), suggesting constraint upon the evolution of thermal limits. Because anthropogenic change is not only shifting global mean temperatures upwards, but also increasing the frequency of weather extremes (Rahmstorf and Coumou, 2011; Ummenhofer and Meehl, 2017), the questions of whether and how species are able to simultaneously adapt to both heat and cold are relevant to their persistence. If the evolution of heat tolerance is constrained by cold tolerance, and vice versa, this may be a major limitation to the ability for adaptation to promote persistence in a more volatile future.

Through encountering novel environments during introduction, NIS present valuable case studies for understanding species responses to rapid environmental change (Huey *et al.*, 2005; Sax *et al.*, 2007). It is possible that some evolutionary divergence of the measured thermal tolerance traits, particularly heat tolerance, occurred prior to introduction through pre-adaptation (see above). However, the high potential for local adaptation in this species and the fact that we observe differentiation of cold performance within a single evolutionary lineage suggests that at least some of the differentiation we observe is driven by rapid evolution within the invasive range. Rapid adaptation in invasive species has been demonstrated in terrestrial, aquatic and marine systems spanning the tree of life (e.g. Maron *et al.*, 2004; Colautti and Barrett, 2013; Sultan *et al.*, 2013; Sotka *et al.*, 2018; Mittan and Zamudio, 2019). These evolutionary shifts suggest that some species/populations have the capacity to rapidly adapt to novel environmental conditions. However, whether or not species contain the adaptive potential necessary to ‘keep up’ with environmental change is an open question (Visser, 2008; Munday *et al.*, 2013; Martin *et al.*, 2023). By investigating how an NIS is able to shift its physiology over contemporary time scales, we present valuable information of the pace of adaptive change in response to novel environmental conditions.

## Data Availability

Physiological data (https://doi.org/10.6084/m9.figshare.25452394.v1) and the code for analysis (https://doi.org/10.6084/m9.figshare.25452403.v1) are publicly available on FigShare.
